# An Uncommon Presentation of Extrahepatic Cholestasis due to Single Biliary Stricture From Kaposi Sarcoma

**DOI:** 10.7759/cureus.8913

**Published:** 2020-06-29

**Authors:** Estefanía Flores Córdova, Madhu Mathew, Hemant Mutneja, Elena Gonzalez Caldito, Melchor Demetria

**Affiliations:** 1 Internal Medicine, Cook County Health and Hospital System, Chicago, USA; 2 Internal Medicine, John H. Stroger, Jr. Hospital of Cook County, Chicago, USA; 3 Gastroenterology and Hepatology, John H. Stroger, Jr. Hospital of Cook County, Chicago, USA

**Keywords:** extrahepatic cholestasis, acquired immunodeficiency syndrome, biliary tract, case report, kaposi sarcoma

## Abstract

Kaposi sarcoma is one of the acquired immunodeficiency syndrome (AIDS) defining diseases. AIDS-associated Kaposi sarcoma affects primarily the skin and the lungs. Although gastrointestinal involvement is relatively common, biliary tract involvement has rarely been reported. It has been associated mostly with extension from liver disease. We describe an uncommon presentation of disseminated Kaposi sarcoma causing extrahepatic cholestasis due to extrahepatic biliary tract involvement that resolved after sphincterotomy with biliary stenting.

We present a case of a 35-year-old African American male diagnosed with human immunodeficiency virus (HIV) infection in 2005. He presented with AIDS after discontinuation of antiretroviral therapy for one year, subsequently being diagnosed with systemic Kaposi sarcoma. He presented with signs and symptoms of obstructive biliary disease, including jaundice, abdominal pain, fatigue, and fever. We encountered a rare presentation of malignant single extrahepatic biliary stenosis secondary to biliary Kaposi sarcoma. The biochemical pattern markedly improved after endoscopic retrograde cholangiopancreatography with sphincterotomy and stenting. However, and despite the resumption of combined antiretroviral therapy, deep immunosuppression caused worsening clinical condition and death five months after initial presentation.

Certainly, among the multiple etiologies of biliary obstruction in AIDS, Kaposi sarcoma is one to consider.

## Introduction

Kaposi sarcoma (KS) is a low-grade vascular tumor associated with human herpesvirus 8 (HHV-8) infection [1,2]. It was first described in 1872 by Moritz Kaposi, a Hungarian dermatologist, initially affecting mostly Mediterranean or Jewish older men. But it was not until 1981 when a demographic change was discovered by Alvin Friedman Kein proving its association with HIV infection [3-5]. There are four variants of its presentation: classic, endemic, posttransplant, and, lastly, the acquired immunodeficiency syndrome (AIDS) associated or epidemic [1,3,5]. The AIDS-associated presentation was for many years the most common AIDS-associated tumor in the United States [6]. Even though KS can develop at any HIV infection stage, generally it occurs in the setting of advanced immune suppression [7]. The incidence has decreased to less than 1% of patients with AIDS after the introduction of combined antiretroviral therapy (cART) [6]. AIDS-associated KS is rapidly progressive and known to initially affect the skin; however, it can extend to mucous membranes and internal organs [1,2,8]. Gastrointestinal involvement is the most common extracutaneous site [2,7]. It is reported that KS can affect any part of the gastrointestinal tract [8]. However, biliary tract involvement has rarely been reported. We present a case with an uncommon presentation of disseminated KS invading the biliary tract and causing extrahepatic cholestasis.

## Case presentation

**Figure 1:**
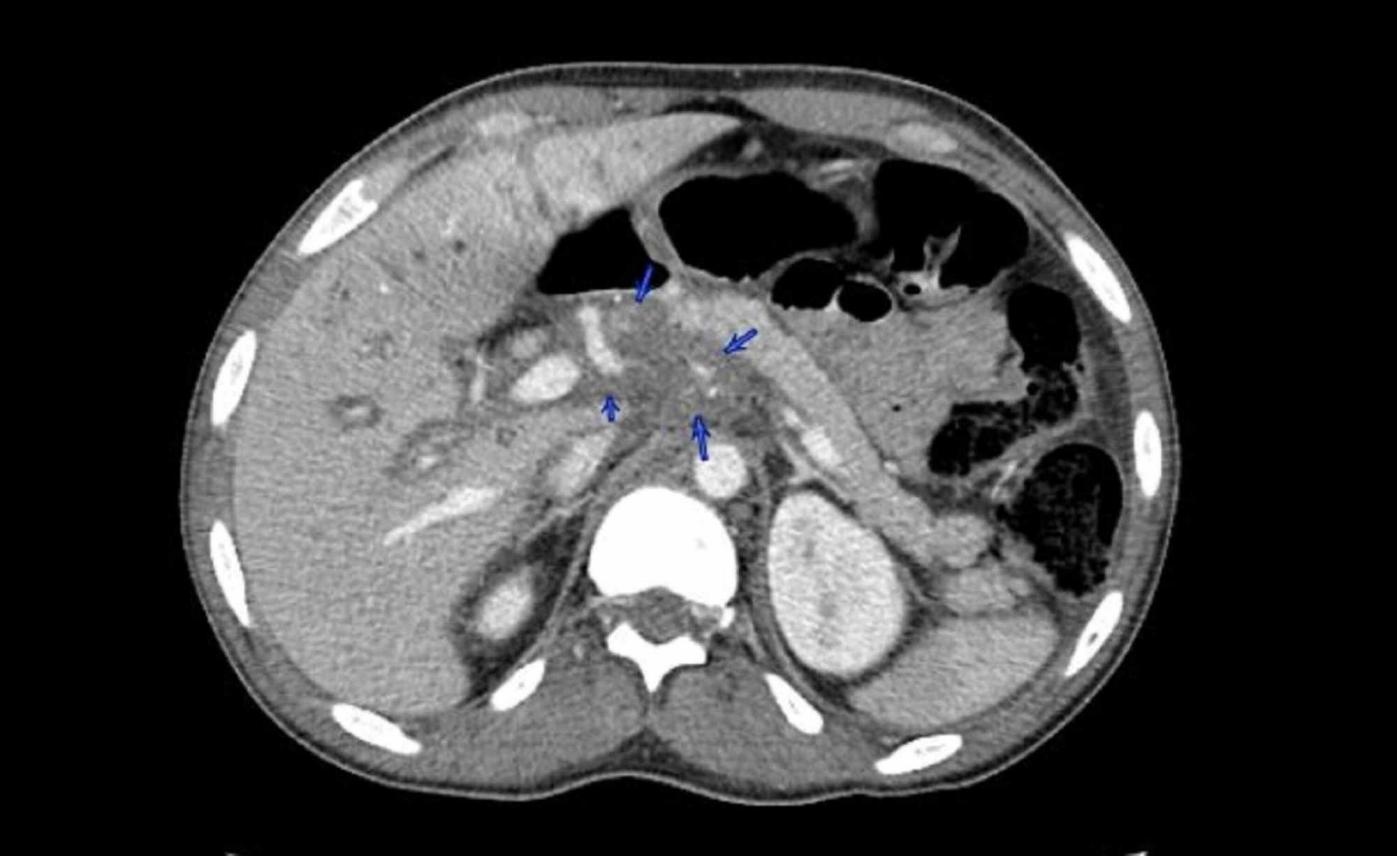
CT of the abdomen CT of the abdomen showing an infiltrative process in the portal hepatis to the celiac area (blue arrows) with obstruction of the biliary tract with intrahepatic biliary dilation. CT, computed tomography

**Figure 2:**
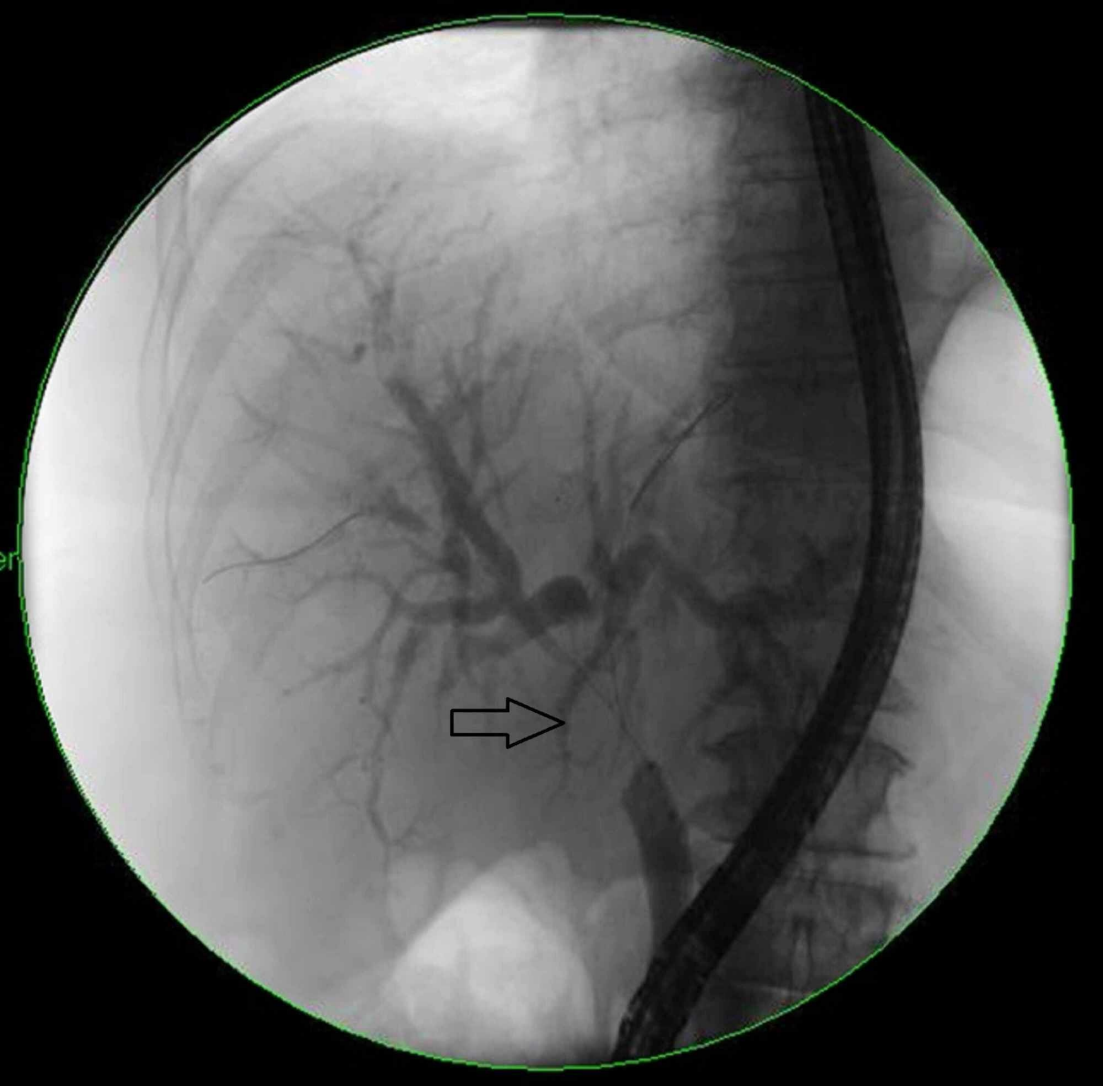
ERCP Common hepatic duct with a 3-cm stricture (black arrow). ERCP, endoscopic retrograde cholangiopancreatography

**Figure 3:**
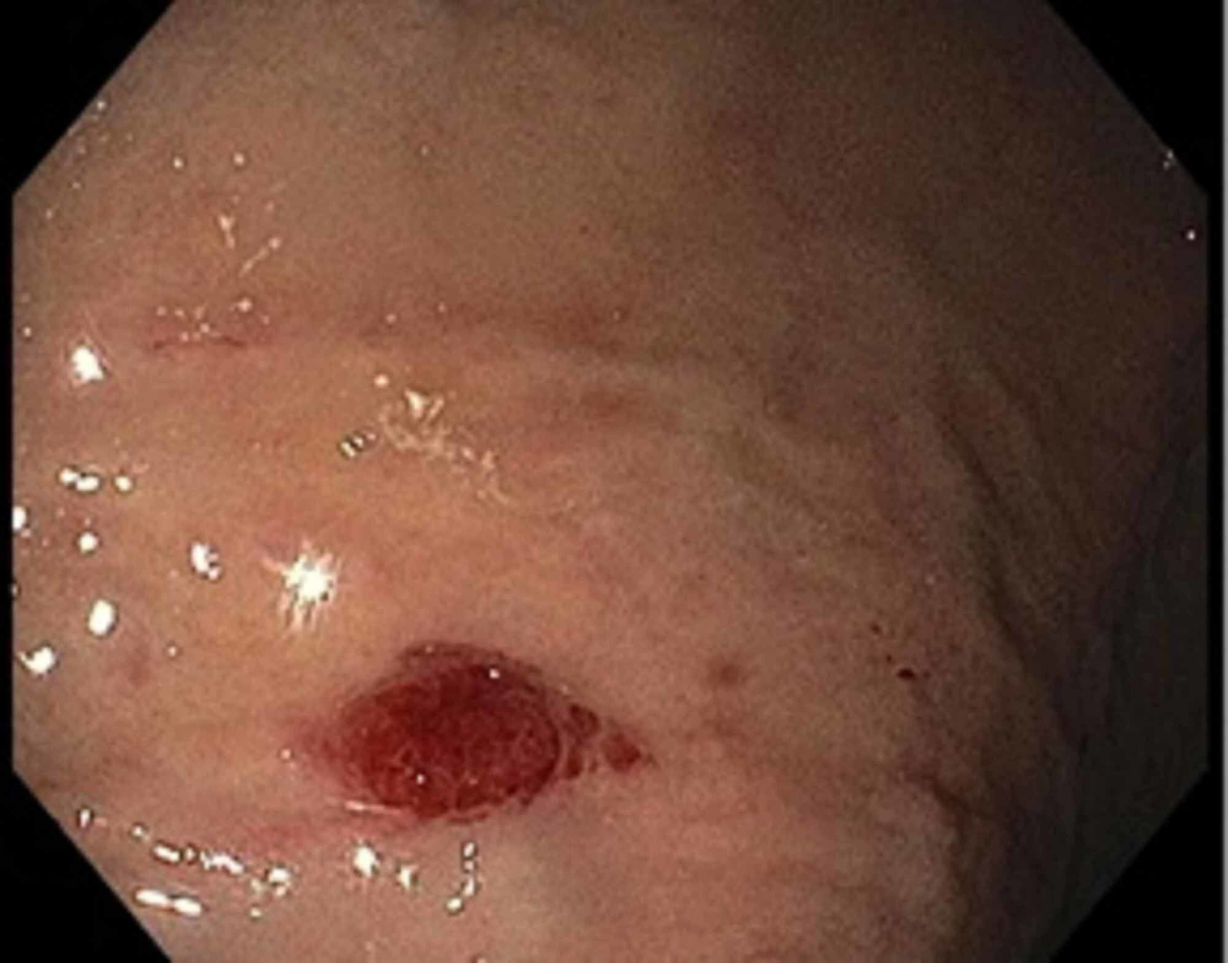
Stomach nodule Erythematous 15-mm nodule in the stomach cardia evidenced during EGD. EGD, esophagogastroduodenoscopy

**Figure 4:**
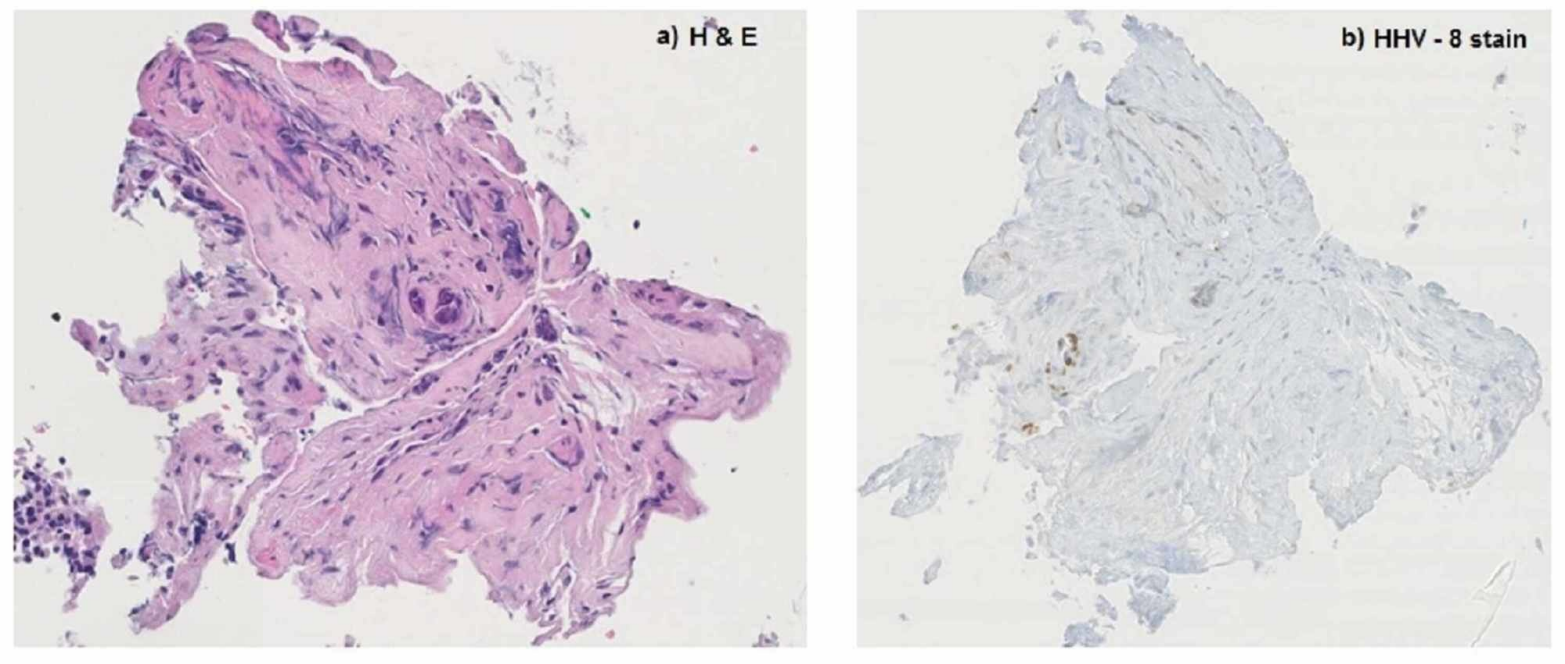
Common hepatic duct biopsy H&E stain (a): benign biliary epithelium and scant fibrous fragments, fibrin, and blood. Immunostain for HHV-8 (b): few endothelial cell nuclei are immunoreactive. H&E, hematoxylin and eosin; HHV-8, human herpesvirus 8

**Figure 5:**
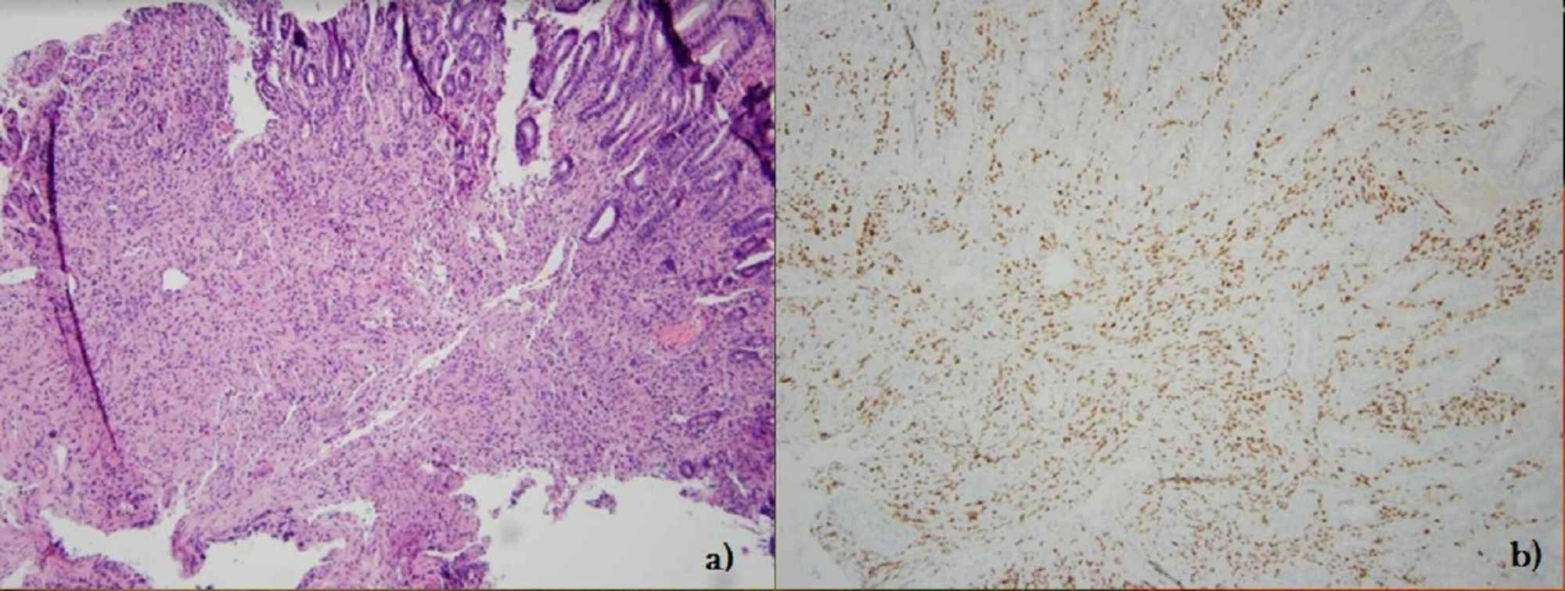
Stomach nodule biopsy H&E stain (a): Kaposi sarcoma involving gastric mucosa. (b): Immunostain for HHV-8 is diffusely positive in the endothelial cell proliferation. H&E, hematoxylin and eosin; HHV-8, human herpesvirus 8

We present the case of a 35-year-old African American male with HIV diagnosed in 2005, initially treated with emtricitabine and tenofovir, although due to lack of insurance he postponed cART for one year. He was admitted to our emergency department with persistent fever, generalized abdominal pain, and jaundice for two weeks and a weight loss of 15 pounds in four weeks. Physical examination on admission showed scleral icterus, small hyperpigmented lesions throughout his trunk, a violaceous nodular enlargement of the hard palate, left axillary lymphadenopathy, a diffusely tender abdomen, and hepatomegaly. Laboratory studies were significant for platelets of 75 k/uL (161-369 k/uL), new-onset conjugated hyperbilirubinemia with a total bilirubin of 23 mg/dL (0.2-1.2 mg/dL), direct bilirubin of 16 mg/dL (0.0-0.2 mg/dL), alkaline phosphatase of 288 IU/L (20-120 IU/L), gamma-glutamyl transferase of 193 IU/L (3-60 IU/L), aspartate aminotransferase of 66 IU/L (0-40 IU/L), alanine aminotransferase of 40 IU/L (5-35 IU/L), and international normalized ratio of 1.3. Computed tomography (CT) of the abdomen revealed hepatomegaly and an ill-defined heterogeneous mass in the peri-portal area with obstruction of the biliary tract with intrahepatic biliary dilation (Figure 1). Endoscopic retrograde cholangiopancreatography demonstrated a non-stenotic erythematous major papilla and a 3-cm stricture in the common hepatic duct (Figure 2). Sphincterotomy and balloon dilation of the stricture were performed, biopsies were taken, and two 7-Fr 12-cm plastic stents were deployed in the right and left hepatic ducts bypassing the stricture. Esophagogastroduodenoscopy with endoscopic ultrasound revealed an erythematous 15-mm nodule in the cardia (Figure 3), with cold biopsies taken and subsequent fine needle biopsy of the peri-portal area performed as well. Inguinal lymph node needle core node biopsy was also performed. Pathology from the porta hepatis mass showed blood clot; however, immunohistochemical staining of the common hepatic duct (Figure 4), stomach nodule (Figure 5), and inguinal lymph node biopsies were positive for HHV-8 KS. cART was commenced, and doxorubicin therapy was started after liver function tests showed improvement. The patient was discharged in view of symptomatic improvement. Liver function tests normalized eight weeks after initial presentation. Nevertheless, his health deteriorated, presenting with hemoptysis, bronchoscopy showed diffuse pulmonary KS, and, subsequently, he had recurrent multiple admission for healthcare-associated pneumonia, cytomegalovirus pneumonitis, pulmonary embolism, and acute tubular necrosis. The patient expired five months after his initial presentation.

## Discussion

Gastrointestinal KS is generally asymptomatic in two-thirds of the cases, and bleeding might be the initial presentation [7,9]. Some patients can present with nonspecific symptoms such as abdominal pain, weight loss, malabsorption, and diarrhea, like the initial clinical presentation of our patient [8,9].

KS involving the biliary tract is infrequent and rarely reported, and an extensive literature review revealed only a few cases. KS has been associated mostly with extension from liver disease presenting with cholangitis and jaundice and may also be caused by extensive disease of the porta hepatis with compression of the extrahepatic biliary tree [10]. A Spanish journal reported a case of biliary tract and gallbladder KS in an AIDS patient without cutaneous involvement that initially presented with pancreatitis; in this case, the involvement of the biliary tract appeared similar to primary sclerosing cholangitis given the beaded pattern [11]. Another study reported a case of a patient with marked changes of sclerosing cholangitis of the intrahepatic bile ducts with postmortem diagnosis of diffuse hepatic KS that caused infiltration of the bile duct [12]. However, our patient had one single 3-cm stricture in the common hepatic duct, which was cannulated with complete resolution of the obstructive pattern. Our patient did not have signs of hepatic involvement nor infiltration extending from the liver. This type of presentation has not been described in the medical literature previously.

Our patient presentation might also be explained by AIDS cholangiopathy, a form of biliary tract inflammation with stricture formation in severely immunosuppressed HIV patients [13,14]. Although the identification of HHV-8 through immunostaining in the tissue biopsy obtained from the biliary tract makes the diagnosis of biliary KS more likely, cutaneous or visceral biopsy is required for the diagnosis of KS. Histopathological features include vascular or spindle cell formations and inflammatory infiltration. Immunohistochemical staining is characteristic of HHV-8 expression, as demonstrated in the common hepatic duct biopsy of our patient [2]. In paraffin-embedded sections, HHV-8 immunostaining has a sensitive (99%) and specific (100%) [8,15].

In patients with advanced symptomatic cutaneous or extracutaneous disease, the role of chemotherapy in addition to standard antiretroviral therapy has been demonstrated to reduce disease progression [2,16]. The current first-line treatment for advanced KS according to JNCCN (Journal of the National Comprehensive Cancer Network) guidelines is liposomal doxorubicin. An alternative option for first-line systemic therapy is paclitaxel [17]. Patients treated with pegylated liposomal doxorubicin or paclitaxel have shown clinical benefit and tumor response, but their side effects are not negligible [16,18]. Newer targeted treatments are being explored; however, the declining incidence of AIDS-related KS makes it difficult to create large-scale trials [19]. HHV-8 infection cannot be eradicated, and complete remission in the setting of advanced disease at presentation is rare; however, reduction or reversion of symptoms and mitigation of end-organ damage can be achieved [17].

As treatment of HIV has improved, a reduction in the incidence of HIV-associated KS has been evidenced; however, in patients without treatment or interrupted therapy, especially those with AIDS, high level of suspicion should be maintained to timely diagnose HHV-8 infection, treat accordingly, and avoid future complications [17,20]. Even though our patient’s symptoms and chemistry improved initially, his marked immunosuppression triggering multiple opportunistic infections caused his death.

## Conclusions

KS is an opportunistic malignancy seen commonly in patients with AIDS and can frequently involve the gastrointestinal tract. Any part of the gastrointestinal tract can be affected by KS. Isolated biliary stricture is rarely reported; however, it must be considered among the multiple etiologies of biliary obstruction in AIDS.
